# Asymptomatic carriage and molecular characterization of *Staphylococcus aureus* in pre-clinical and clinical medical students

**DOI:** 10.1007/s12223-024-01237-1

**Published:** 2025-01-13

**Authors:** Kristýna Brodíková, Bohdana Rezková, Ivana Koláčková, Renáta Karpíšková

**Affiliations:** Department of Public Health, Faculty of Medicine, Masaryk University, Kamenice 753/5, 625 00 Brno, Czech Republic

**Keywords:** *Staphylococcus aureus*, MRSA, Medical students, *Spa* type, Antibiotic resistance, Virulence genes

## Abstract

Medical students are exposed to the hospital environment and patients during their studies, increasing the risk of exposure to virulent and antibiotic-resistant isolates of *Staphylococcus aureus*. The aim of the study is to determine the prevalence of *Staphylococcus aureus* among medical students who have varying levels of exposure to the hospital environment to provide valuable insights into the risk of colonization and transmission. Nasal swabs and fingerprints were obtained and cultured on a selective medium for staphylococci. The obtained isolates were confirmed as methicillin-sensitive *S. aureus* (MSSA) or methicillin-resistant (MRSA) using PCR. Antibiotic resistance, the presence of virulence genes including enterotoxin encoding genes, and *spa* typing were performed. Among pre-clinical students, MSSA was detected on the nose in 45.2% and on the fingerprints in 10.6% of the participants. Among clinical students, MSSA was detected on the nose in 42.0% and on the fingerprints in 25.4%. Only one MRSA isolate was obtained. Genes *seg* and *sei* were the most frequently detected in both student groups, with their presence in over 40% of isolates among clinical students. The *eta* and *etb* genes were mainly detected from the nose in both student groups. In pre-clinical students, *S. aureus* carrying *eta* gene occurred in 6.4% and *etb* in 8.5%. In clinical students, the occurrence was 5.1% for *eta* and 8.5% for *etb*. The *tst* gene was identified only in the nose and fingerprints of the clinical student group. The most frequently observed resistance was to clindamycin and erythromycin. In total 58 different *spa* types were identified. High rates of asymptomatic MSSA carriage were observed in both groups of medical students. Detected MSSA strains showed a high degree of genetic variability, with a number of them carrying the virulence and antibiotic resistance genes. Although students do not exhibit increased risk to their patient’s, increased hygiene is required in asymptomatic carriage personnel. The overall prevalence of MRSA was low, with a minimal risk of spread.

**Supplementary Information:**

The online version contains supplementary material available at 10.1007/s12223-024-01237-1.

## Introduction

*Staphylococcus aureus* (Sa) is a well-known cause of infections but also a common inhabitant of the human body. Approximately 20–30% of the healthy human population can be asymptomatically colonized by Sa, with the most common colonization sites being the nasal mucosa and nasopharynx (Centers for Disease Control and Prevention 2019; Stensen et al. 2022). Sa infections can arise from carriage, and there is also the risk of transmitting Sa to more susceptible individuals, leading to dermatitis, abscess formation, or serious systemic bloodstream diseases (McCaig et al. 2006; Pomorska et al. 2021).

Treating Sa infections can be challenging, particularly in cases involving methicillin-resistant isolates of Sa (MRSA). MRSA can originate from various sources. Community-associated MRSA (CA-MRSA) occurs within communities of individuals with close contact, such as athletes, military personnel, school students, or people sharing households. Transmission to pets is also possible (Centers for Disease Control and Prevention 2019; Zhu et al. 2021).

Sa is the second most common causative agent of healthcare-associated infections overall and is the most common cause of surgical site infections (European Centre for Disease Prevention and Control 2023a). Hospital-acquired MRSA (HA-MRSA) is associated with the hospital environment and can be acquired through exposure to infected patients or during hospital stays and surgeries (Centers for Disease Control and Prevention 2024). Livestock-associated MRSA (LA-MRSA) is found among livestock animals and in food products of animal origin (Anjum et al. 2019).

There is a significant risk of asymptomatic carriage of virulent isolates of Sa including MRSA among medical personnel who have frequent contact with patients and the hospital environment. According to the CDC, approximately 2% of people are colonized by MRSA without developing infection (Centers for Disease Control and Prevention 2019). A systematic review conducted on hospital personnel in Europe and the USA estimated the pooled MRSA colonization rate to be 1.8%. Furthermore, there are substantial variations in the prevalence of invasive MRSA across European countries with a gradient of increasing prevalence from north to south (Dulon et al. 2014; European Centre for Disease Prevention and Control 2023b). Medical students also face risks, as they are intensively exposed to the hospital environment during their studies and come into direct contact with patients. Studies confirm that an extended duration of stay in hospital facilities by medical students increases the risk of MRSA carriage as well as methicillin-sensitive Sa (MSSA) (Arıkan et al. 2021; Bhatta et al. 2018).

The transmission of Sa is influenced by the level of hygiene, particularly hand hygiene. Sa isolates, including MRSA, are commonly found on frequently touched surfaces, not only in the hospital environment. These isolates have also been detected in the surroundings of university campuses, public transport, and shopping baskets in supermarkets (Domon et al. 2015; Lutz et al. 2014; Thapaliya et al. 2017).

A study from the Czech Republic conducted in 2012–2013 in medical students from the Olomouc region showed MSSA carriage in 61 of 206 (29.6%) first-year medical students and 32 of 101 (32.5%) fifth-year general medicine students. MRSA was not detected (Holy et al. 2015). However, a more recent study conducted in 2021 from the city of Prague shows that the colonization rate in students was almost 45%, of which 2 isolates (1.1%) were characterized as MRSA. Of the 178 students involved in the study, 118 belonged to the first year and 60 to the sixth year, with no significant difference in carriage between the groups. MRSA was detected in both groups (Smelikova et al. 2023). Despite the similar methodological approach, the study produced different prevalence results.

The aims of the study are as follows: (i) to determine the prevalence of Sa among medical students who have varying levels of exposure to the hospital environment, (ii) to assess the prevalence of methicillin-resistant Sa (MRSA) among medical students, (iii) to characterize the obtained isolates of Sa, including their virulence factors and antibiotic resistance profiles, (iv) to provide valuable insights into the risk of Sa colonization and transmission among medical students and the potential implications for patient care and infection control measures.

## Materials and methods

### Subject and sample collection

The study was approved by the Faculty of Medicine Ethics committee at Masaryk University with approval number 34/22 and it was conducted in the pre-clinical (March 2022–June 2022) and in the clinical student groups (October 2022–January 2023). The pre-clinical group of students was in the 3rd year, while the clinical students were in the 5th year of their study. Informed consent was obtained from each participating student.

The study included pre-clinical and clinical medical students within the age range of 20–25 years. Pre-clinical medical students represented the unexposed group, while clinical medical students were exposed to the hospital environment.

Two types of samples were collected from each participant: nasal swabs were collected from the anterior vestibule using dry cotton swabs. The swabs were then inserted into Amies transport medium (COPAN, Italy). Fingerprints of the forefinger and thumb of the dominant hand were collected on Baird-Parker medium (BP; Biokar Diagnostics, France). The BP plates were incubated at 37 °C 48 h. Nasal swab specimens were pre-enriched in 3 ml of buffered peptone water (Oxoid, UK) and incubated at 37 °C 18–24 h. After pre-enrichment the enriched swabs were streaked on the following media; BP and chromatic MRSA (Liofilchem, Italy). The presence and characteristics of bacterial growth on each plate were recorded.

### MSSA and MRSA identification

The identification of Sa and MRSA isolates were performed using multiplex PCR. All suspected colonies were tested for the presence of Sa-specific fragment SA-442 (Martineau et al. 1998) and the *mecA* gene (Oliveira and de Lencastre 2002), which is responsible for methicillin resistance. The presence of the *mecC* gene, also responsible for methicillin resistance, was not tested.

### Virulence gene detection

All isolates confirmed as Sa were further tested using the PCR method for the presence of genes for the presence of virulence genes. The following genes were investigated: enterotoxins (*sea*, *seb*, *sec*, *sed*, *see*, *seg*, *seh*, *sei*, *sej* (Løvseth et al. 2004; Monday and Bohach 1999), exfoliative toxins (*eta*, *etb*) (Hososaka et al. 2007), Panton-Valentine leucocidin (*pvl*) (Lina et al. 1999), and toxic shock syndrome toxin (*tst*) (Mehrotra et al. 2000).

PCRs were performed with PPP Master Mix (Top-Bio, Czech Republic), except for the detection of enterotoxins, where a polymerase from Qiagen (Germany) was used. Primers prepared by Generi Biotech (Czech Republic) were used for all PCRs. PCR results were visualized by electrophoresis on a 2% agarose gel (BioConcept, Switzerland) with the addition of the fluorescent dye Midori Green Advance (NIPPON Genetics, Germany).

### Antibiotic resistance

Sa isolates were tested for antimicrobial resistance using the disk diffusion method with antibiotic disks from Oxoid (UK) on a Mueller–Hinton medium (Oxoid, UK). The following panel of antibiotics was used for testing: DA, clindamycin (2 µg); E, erythromycin (15 µg); CN, gentamicin (10 µg); TE, tetracycline (30 µg); C, chloramphenicol (30 µg); CIP, ciprofloxacin (5 µg); FOX, cefoxitin (30 µg); RD, rifampicin (5 µg). MRSA isolate was also tested for FD, fusidic acid (10 µg), and MUP, mupirocin (200 µg). The results were interpreted according to the guidelines provided by the European Committee on Antimicrobial Susceptibility Testing 2023.

The D-test was performed to detect the inducible type of resistance (iMLS) to erythromycin and clindamycin. During testing, antibiotic disks DA (2 µg) and E (15 µg) were placed adjacent to each other. In the presence of iMLS resistance, a D-shaped zone of inhibition was observed around the DA disk (Sasirekha et al. 2014).

### Spa typing and multilocus sequence typing (MLST)

Polymorphisms in the X region of the gene for protein A (*spa*) were detected by PCR followed by sequencing (Strommenger et al. 2008). The PCR products were purified using the Roche purification kit (Germany), and the products were externally subjected to Sanger sequencing by Eurofins genomics (Germany). The obtained sequences were evaluated using the online tool at https://spatyper.fortinbras.us/. Sequence comparisons and the construction of a phylogenetic tree were performed using MEGA11: Molecular Evolutionary Genetics Analysis version 11 (Tamura et al. 2021).

Purification and sequencing of the MRSA isolate were performed similarly to *spa* typing (Enright et al. 2000). Multi-locus sequence typing MLST was carried out using previously reported primers for seven housekeeping genes: *arcC*, *aroE*, *glpF*, *gmk*, *pta*, *tpi*, and *yqiL*. The obtained sequences were compared using Blastn, and the allele for each gene was determined. The allelic profile was entered into PubMLST (Jolley et al. 2018) to determine the sequence type.

### Statistical analysis

The chi-square test was used for statistical comparison. A *p*-value of *p* < 0.05 was considered statistically significant.

## Results

### MSSA and MRSA prevalence

In the study, a total of 104 pre-clinical and 138 clinical students were enrolled. No nasal MRSA carriage was detected in the pre-clinical group. However, one MRSA isolate was obtained from a female student in the clinical group (Table 1.). When examining hand fingerprints, a lower number of MSSA isolates were compared to a nasal carriage. No MRSA isolates were detected from the hand fingerprints of the students in either group.

**Table 1:** MSSA and MRSA prevalence among the pre-clinical and clinical students categorized by body parts (nose and fingerprints)

Sample type	Resistance to methicillin	Group of students	
		Pre-clinical	Clinical
Nose	MSSA	47/104 (45.2%)	58/138 (42.0%)
	MRSA	0/104 (0.0%)	1/138 (0.7%)
Fingerprints	MSSA	11/104 (10.6%)	35/138 (25.4%)
	MRSA	0/104 (0.0%)	0/138 (0.0%)

Positive-to-total, percentage in parentheses: The first number are the positive samples, the second are total samples, and the percentages are the positive-to-total ratio in parentheses.

### Characterization of virulence genes

Among both groups of students and in both types of samples, the most frequently detected genes encoding enterotoxins were *seg* and *sei*. In the pre-clinical student group, *seg* occurred in 19.1% of nasal isolates and 27.3% of fingerprint isolates. Similarly, *sei* was found in 21.3% of nasal swab isolates and 27.3% of fingerprint isolates. In the clinical student group, the prevalence of *seg* and *sei* in nasal swab isolates was even higher, reaching 40.0%. For fingerprint isolates, *sei* was detected in 42.7% of cases, while *seg* was present in 20.0% of cases. Furthermore, in the clinical student group, additional genes encoding enterotoxin production, namely *sea* and *seb* were found. These genes were not detected in the pre-clinical student group.

Isolates from both groups of students showed positive results for the presence of *eta* and *etb* genes. In the pre-clinical student group, the genes were found exclusively in nasal swab isolates, with *eta* being present in 6.4% and *etb* in 8.5% of the isolates. Among the nasal swab isolates of clinical students, *eta* was detected in 5.1% of the isolates, and *etb* was present in 8.5%. Interestingly, the *etb* gene was only detected in the fingerprints of clinical students, accounting for 5.7% of the isolates. Furthermore, the *tst* gene was identified in isolates from the nose and fingerprints of clinical students, with a prevalence of 8.5% and 17.1% respectively. However, no isolate carrying the *pvl* gene was confirmed in either group.

### Antibiotic resistance detection

All the MSSA and MRSA isolates obtained were subjected for antibiotic resistance screening. The results revealed that the most frequent resistance was observed against both erythromycin and clindamycin. Among the nasal isolates, approximately 34.0% of isolates from pre-clinical students and 23.7% of isolates from clinical students exhibited resistance to erythromycin. Similarly, 23.7% of nasal isolates from pre-clinical students and 22.0% of nasal isolates from clinical students showed resistance to clindamycin.

Notably, among the nasal isolates from pre-clinical students, a significant proportion (29.8%) exhibited inducible clindamycin resistance (iMLS). This trend was also observed in the nasal isolates from clinical students, with 22.0% of isolates showing the iMLS type when resistance to erythromycin and clindamycin was detected. Furthermore, a smaller percentage of isolates displayed constitutive resistance (cMLS) to erythromycin and clindamycin. Specifically, two (4.2%) isolates from nose swabs of pre-clinical students and one (1.7%) isolate from the nose swabs of clinical students exhibited the cMLS type of resistance.

Among the clinical students, the most frequently observed resistance was to ciprofloxacin, with 22.0% of isolates from the nose and 20.0% of isolates from fingerprints showing resistance to this antibiotic. In contrast, resistance to ciprofloxacin was present in only 2 isolates (4.3%) obtained from nasal swabs of pre-clinical students, resistance to chloramphenicol was found in 3 isolates (1.2%) obtained from all students, and resistance to tetracycline was observed specifically from the clinical students.

A multidrug resistance, defined as resistance to three or more antibiotic groups, was recorded in two isolates. It was found only in the group of clinical students. Both two cases involved resistance to erythromycin and clindamycin, in combination with chloramphenicol. Regarding the MRSA isolate, resistance to cefoxitin was detected, confirming its resistance to methicillin. Resistance to fusidic acid was observed, while resistance to mupirocin was not detected. More detailed information on virulence and antibiotic-resistance genes is presented in Supplementary Table 1.

### *Spa* typing and MLST

Concurrent capture of MSSA from both the nose and fingerprints was observed in 10 out of 104 pre-clinical students (9.6%) and in 28 out of 138 clinical students (20.3%). In 60% of pre-clinical students, the same *spa* type was identified in isolates from both the nose and fingerprints. Among clinical students, this was observed in 32.1% of cases.

An interesting finding is that spa type t10060 was frequent (12.1%) in the preclinical group, whereas it has been reported once (0.7%) in the clinical group. Other more frequently represented *spa* types among the pre-clinical students were t084 (8.6%), t085 (6.9%), t078 (5.2%), and t267 (5.2%). The spectrum of *spa* types varied between both student groups. For the clinical group, the five most common *spa* types were t20949 (5.3%), t491 (5.3%), t078 (4.3%), t1451 (4.3%), and t179 (4.3%). More information on the frequency of each *spa t*ype is provided in Supplementary Table 1. The MRSA isolate belonged to *spa* type t051. A total of 15 (14.2%) nasal swab isolates and 12 (26.1%) fingerprint isolates could not be assigned to the *spa* type, these sequences are not found in the available database.

Molecular typing of the MRSA isolate obtained from a female student in the clinical student group revealed it to be assigned to sequence type ST2149, belonging to the clonal complex CC8.

**Fig. 1:**
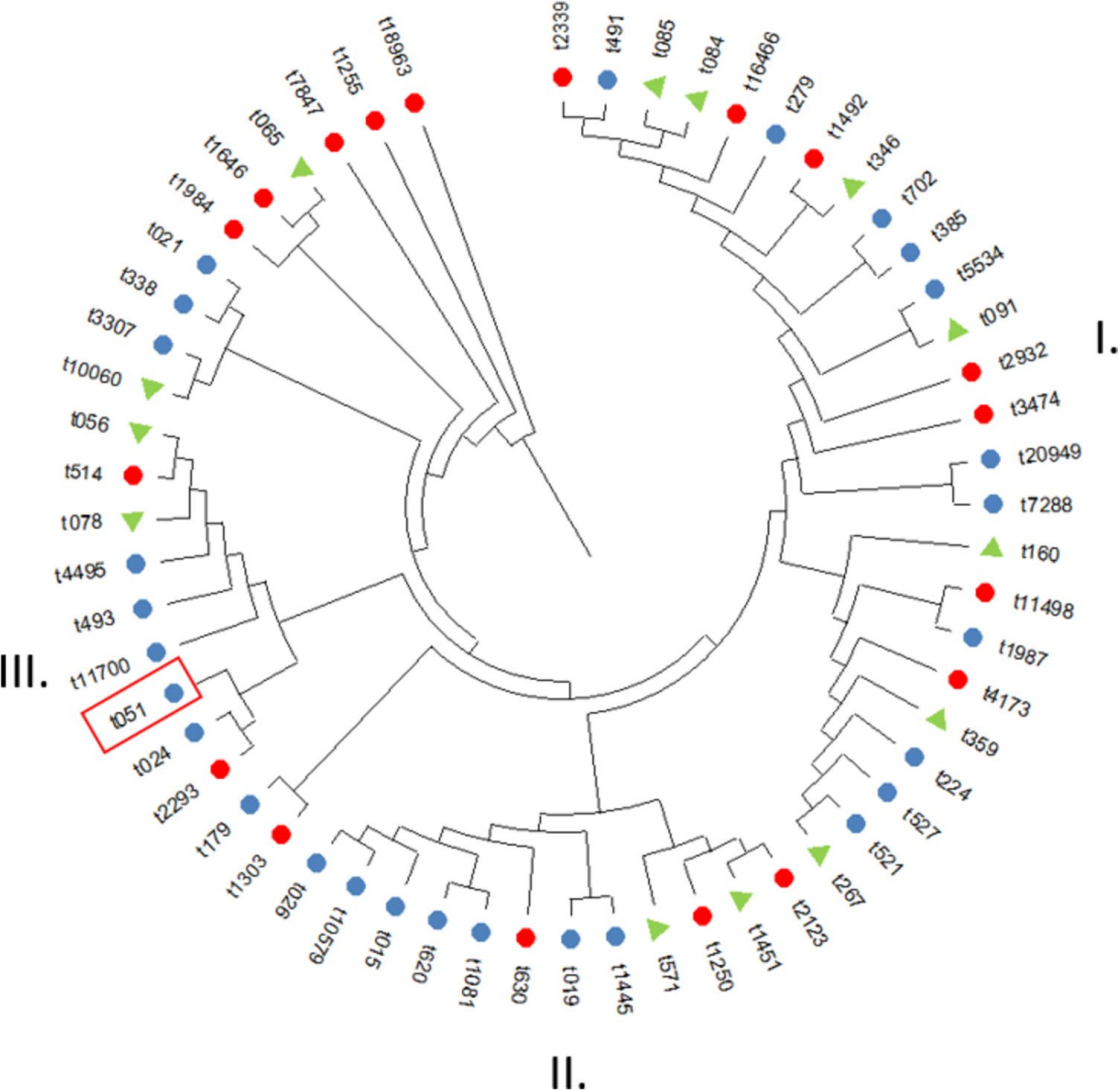
The evolutionary history was inferred using the UPGMA method (Tamura et al. 2004). The optimal tree is shown. The evolutionary distances were computed using the Maximum Composite Likelihood method and are in the units of the number of base substitutions per site. This analysis involved 55 nucleotide sequences. All ambiguous positions were removed for each sequence pair (pairwise deletion option). There was a total of 584 positions in the final dataset. Evolutionary analyses were conducted in MEGA11 (Tamura et al. 2021). Red pre-clinical students, blue clinical students, Green—both groups

The phylogenetic tree of all recovered *spa* types is shown in Fig. 1. A total of 58 different *spa* types were detected among the obtained isolates. The *spa* types were differentiated into 6 clusters, the larger clusters including the most frequently occurring *spa* types were labeled I., II., and III. Eighteen different *spa* types were obtained from a group of pre-clinical students (red), and 27 were obtained from clinical students (blue). Common *spa* types for both groups were recorded 13 (green). The MRSA isolate is highlighted by the red frame.

## Discussion

Healthcare professionals and medical students are considered to be at higher risk of carrying MSSA as well as MRSA. For medical students, this risk increases with longer stays in hospital facilities during mandatory internships. Although the prevalence of MSSA may increase with the length of exposure to the hospital environment, we did not observe a significant difference in the nasal prevalence of MSSA between our groups of students (*p* = 0.623) as well as other authors (Arıkan et al. 2021; Sharma et al. 2020).

The prevalence of MSSA in both groups of students exceeded the reported rates in other studies, where nasal carriage of Sa ranged from 20 to 30% (Chen et al. 2012; Gualdoni et al. 2012). The higher prevalence observed in this study may be attributed to the methodology used for sample processing. Studies that employed direct culture without pre-enrichment reported lower rates of MSSA detection, whereas those utilizing enrichment techniques demonstrated higher rates (Sarkar et al. 2016; Stensen et al. 2022). Stensen et al. (2002) compared the recovery of Sa using direct culture and using enrichment culture, identifying Sa in 30.3% of samples using direct culture and 42.6% using enrichment culture (Stensen et al. 2022). These findings underscore the impact of different laboratory methods on the observed prevalence rates and highlight the need for standardized protocols in future studies.

Interestingly, the incidence of MSSA from fingerprints was significantly higher (*p* = 0.004) in the clinical student group compared to the pre-clinical one. This difference may be attributed to variations in exposure to environmental contamination, frequency of contacts and other behavioral factors or, more likely, differences in attitudes towards hand hygiene This finding emphasizes the ongoing need for reinforcing the importance of proper hand hygiene practices among medical students throughout their education.

The presence of MRSA isolates among medical students is a concerning finding, as it suggests potential colonization even before exposure to the hospital environment or gradual acquisition over time (Orlin et al. 2017). In our study, MRSA was detected in one of 138 students enrolled in the clinical group. This is less than reported by a study focusing on hospital personnel in Europe and the USA (Dulon et al. 2014). The low prevalence may be related to the current epidemiological situation and the declining trend of MRSA incidence in the country. Within Europe, the Czech Republic is among the countries with a lower MRSA incidence and a gradual decline was reported in recent years (European Centre for Disease Prevention and Control 2023b). The MRSA isolate identified in this study was not a multidrug-resistant strain but exhibited resistance to fusidic acid, which is commonly used for decolonization purposes. The resistance to fusidic acid raises concern regarding potential treatment failures (Banerjee and Argáez 2017). The student carrying the MRSA isolate had no risk contacts and had not recently taken antibiotics. During her compulsory practice, she visited the Department of Pulmonary Diseases and was in contact with patients with tuberculosis. The CC8 to which MRSA isolate belonged is frequently circulating in Czech hospitals, suggesting a possible acquisition during the student´s stay in a hospital environment (Pomorska et al. 2021).

Enterotoxins *seg* and *sei* dominated in both groups of students, with a significant difference in nasal swab isolates. The clinical student group, with higher exposure to the hospital environment, exhibited a higher prevalence of *sei* (*p* = 0.034) and *seg* (*p* = 0.017) genes compared to the pre-clinical group. These enterotoxins lay close to each other on the chromosome and are therefore often found together in the same isolate (Jarraud et al. 1999). Enterotoxins *seg* and *sei* are also associated with toxic shock syndrome and scarlet fever. This was also confirmed by our results, where all isolates from both groups that carried *eta* or *etb* simultaneously carried *seg* and *sei* genes. *tst* genes were demonstrated only in clinical students with a significant difference (*p* = 4.42E-10) compared to pre-clinical students. All isolates obtaining the *tst* gene simultaneously carried only the *seg* gene and not the *sei* gene (Jarraud et al. 1999).

In terms of antibiotic resistance, the majority of isolates were found to be sensitive to tested antibiotics. However, resistance to erythromycin and clindamycin, particularly the inducible MLS resistance type, was prevalent among MSSA isolates in both groups. Sa with inducible MLS resistance, appear to be sensitive to clindamycin, but due to the presence of *erm* genes, they are resistant to this type of antibiotic. This finding is of clinical significance as clindamycin is an important antibiotic for treating staphylococcal infections, including MRSA, and its efficacy may be comprised in the presence of inducible resistance (Sasirekha et al. 2014).

The analysis of *spa* types provided insights into the genetic diversity of Sa isolates among the student groups. A total of 58 different *spa* types were identified among the 152 isolates, indicating a high variability of *spa* types within the MSSA circulating among the students. The generated phylogenetic tree shows the mutual relatedness based on the *spa* type. Six clusters were formed, with the three larger clusters indicated by numbers in Fig. 1. Interestingly, the 5 most frequently detected *spa* types in both groups occurred in the largest cluster I, suggesting that the most prevalent MSSA isolates are more closely related to each other. In contrast, the most frequent *spa* type common to both groups of students, t078, occurred in cluster III, farther away from the other frequent *spa* types. Among clinical isolates in Europe, t032, t002, and t008 are frequently detected (Asadollahi et al. 2018), but they were not recorded in any group of students in this study. It is worth noting that 31 isolates could not be assigned a *spa* type, suggesting the novel or uncharacterized *spa* types not yet recorded in the available database. This highlights the need for continuous surveillance and updating of databases to accommodate emerging strains and ensure accurate identification and characterization of Sa isolates.

## Conclusions

In conclusion, this study provides insights into the prevalence and characterization of MSSA and MRSA among medical students. By including data on virulence, resistance, and isolate typing, it addresses critical aspects that are often overlooked in similar research. This could provide a more holistic understanding of the subject matter. We did not find that the medical students in our cohort were significant source of MRSA to their patients and the general population. However, the high prevalence of MSSA, the presence of MRSA, and the diversity of *spa* types observed among the student groups warrant further research to better understand the epidemiology and dynamics of Sa infections in this population. Such knowledge will contribute to the development of targeted interventions to prevent and control the spread of Sa in healthcare environments.

## Supplementary Information

Below is the link to the electronic supplementary material.

Supplementary file1 (XLSX 26 KB)

## Data Availability

The data underlying this article will be shared on reasonable request to the corresponding author.

## Abbreviations

*BP*: Baird-Parker medium
*CA-MRSA*: Community-associated MRSA
*C*: Chloramphenicol
*cMLS*: The constitutive resistance to erythromycin and clindamycin
*CIP*: Ciprofloxacin
*CN*: Gentamicin
*DA*: Clindamycin
*E*: Erythromycin
*eta**, **etb*: Exfoliative toxins
*FD*: Fusidic acid
*FOX*: Cefoxitin
*HA-MRSA*: Hospital-acquired MRSA
*iMLS*: The inducible type of resistance to erythromycin and clindamycin
*LA-MRSA*: Livestock-associated MRSA
*MLST*: Multilocus sequence typing
*MRSA*: Methicillin-resistant *Staphylococcus aureus*
*MSSA*: Methicillin-sensitive *Staphylococcus aureus*
*MUP*: Mupirocin
*pvl*: Panton-Valentin leucocidin
*RD*: Rifampicin
*Sa*: *Staphylococcus aureus*
*spa*: Polymorphisms in the X region of the gene for protein A
*TE*: Tetracycline
*tst*: Toxic shock syndrome toxin
